# Supplementary material for: Mobilization of Endogenous CD34+/CD133+ Endothelial Progenitor Cells by Enhanced External Counter Pulsation for Treatment of Refractory Angina
Source: Int J Mol Sci. 2024 Sep 18;25(18):10030. doi: 10.3390/ijms251810030 (PMC11432706; doi:10.3390/ijms251810030)
Supplement: Supplementary file 1 [file ijms-25-10030-s001.zip › Table S1 Effect of baseline clinical variables on MACE hazard.pdf]

Table S1. Cox Regression analysis of baseline clinical characteristics of RA patients treated with EECP or cardiac rehabilitation for effects on MACE hazard

**Clinical characteristic**	**Value**		***p*-values of effect on MACE**			
Continuous variables	*Mean ± SD*		*Univariate*		*Multivariate**	
	EECP	Rehab	EECP	Rehab	EECP	Rehab
Age	68±10	66±7 yrs	0.71	0.90	-	-
Body mass index (BMI)	29.8±5.3	29.6±7.4	0.14	0.47	0.09	-
High density lipids (HDL)	40.2±8.1	46.7±10.5	0.69	0.36	-	-
Low density lipids (LDL)	75.3±19.4	94±26.5	0.16	0.11	0.22	0.34
Left ventricular ejection fraction (LVEF)	0.44±0.14	0.57±0.09	0.39	0.11	-	0.97
Categorical variables	*(% with)*		*Univariate*		*Multivariate**	
Antithrombin	24	9	0.40/	0.55	-	-
Calcium channel blocker	38	27	0.25	0.34	-	-
Coronary artery bypass graft	63	36	0.41	0.23	-	-
Clopidogrel	40	45	0.91	0.22	-	-
Diabetes	28	36	0.46	0.12	-	0.19
Diuretic	38	36	0.66	0.66	-	-
Former smoker	46	55	0.25	0.97	-	-
Hypertension	62	73	0.44	0.97	-	-
Nitrate	49	9	0.16	0.59	0.98	-
Percutaneous transluminal coronary angioplasty	39	64	0.31	0.23	-	-
Pentoxiphylline	19	0	0.62	-	-	-

*Clinical variables with *p*-values <0.20 in univariate Cox regression tests were entered into a multivariate 1-way regression model to test for significant confounder effects on MACE hazard. *p*-values <0.05 were considered significant.
