# Supplementary material for: Mobilization of Endogenous CD34+/CD133+ Endothelial Progenitor Cells by Enhanced External Counter Pulsation for Treatment of Refractory Angina
Source: Int J Mol Sci. 2024 Sep 18;25(18):10030. doi: 10.3390/ijms251810030 (PMC11432706; doi:10.3390/ijms251810030)
Supplement: Supplementary file 1 [file ijms-25-10030-s001.zip › Table S2 Patient MACE and Cell Count Data.pdf]

Patient #	Trtmt	Responded to therapy	Time to Event (Months)	MACE	Log CD133 counts 1st Half	Log CD133 counts 2nd Half	% Δ log CD133 counts	Log KDR counts 1st Half	Log KDR counts 2nd Half	% Δ log KDR counts	Log CD34 counts 1st Half	Log CD34 counts 2nd Half
1	EECP	no	64	yes, stent	1.053	1.041	-1.1	1.754	1.900	8.4	2.364	2.420
2	EECP	yes	97	no	n/a	n/a	n/a	n/a	n/a	n/a	n/a	n/a
3	EECP	no	22	yes, de novo lesion	1.775	1.916	8	2.580	2.125	-17.6	2.588	2.792
4	EECP	no*	1	no, withdrew from study	1.911	1.792	-6.2	n/a	n/a	n/a	2.583	2.709
5	EECP	no	42	yes, myocardial infarction	1.580	1.903	20.5	n/a	n/a	n/a	2.934	2.969
6	EECP	no	84	yes, LAD stent	1.628	1.924	18.2	n/a	n/a	n/a	3.017	3.182
7	EECP	no	82	no	1.727	1.431	17.1	n/a	n/a	n/a	3.612	2.723
8	EECP	no	87	no	1.620	1.462	-9.7	1.349	1.482	9.9	2.653	2.624
9	EECP	yes	17	yes, stent	1.467	1.769	20.6	1.523	2.031	33.4	2.873	2.985
10	EECP	yes	86	no	1.728	2.013	16.5	1.616	1.903	17.7	2.933	2.939
11	EECP	no	9	yes, stent	n/a	n/a	n/a	n/a	n/a	n/a	n/a	n/a
12	EECP	yes	79	no	1.574	2.316	47.1	1.628	2.137	31.2	2.631	2.741
13	EECP	no	72	yes, LAD stent	1.756	1.908	8.7	2.035	2.674	31.4	3.257	2.936
14	EECP	yes	75	no	2.060	2.188	6.2	2.167	2.256	4.1	2.973	2.752
15	EECP	yes	53	yes, died of heart failure	1.797	2.202	22.6	1.988	2.139	7.6	2.644	2.531
16	EECP	yes	69	no	n/a	n/a	n/a	n/a	n/a	n/a	n/a	n/a
17	EECP	no	34	yes, de novo lesion	2.037	1.782	-12.5	1.853	1.799	-2.9	3.209	3.365
18	EECP	no	35	yes, died of heart failure	2.069	2.436	17.7	2.232	2.688	20.4	4.418	4.511
19	EECP	yes	36	yes, pacemaker for heart failure	2.365	2.567	10.5	2.728	2.729	0.6	4.546	4.349
20	EECP	no	13	yes, died of heart failure	n/a	n/a	n/a	n/a	n/a	n/a	n/a	n/a
21	EECP	yes	62	no	n/a	n/a	n/a	n/a	n/a	n/a	n/a	n/a
22	EECP	no	7	yes, hospitalized for heart failure	n/a	n/a	n/a	n/a	n/a	n/a	n/a	n/a
23	EECP	yes	12	yes, restenosis	n/a	n/a	n/a	n/a	n/a	n/a	n/a	n/a
24	EECP	yes	58	no	n/a	n/a	-15.9	n/a	n/a	-23	n/a	n/a
25	EECP	no	54	no	0.699	1.230	76	2.332	1.606	-31.2	3.127	3.383
26	EECP	yes	52	no	n/a	n/a	n/a	n/a	n/a	n/a	n/a	n/a
27	EECP	no	3	yes, unstable angina	n/a	n/a	0	n/a	n/a	9.8	n/a	n/a
28	EECP	no	24	yes, died of heart failure	1.337	1.451	8.5	0.602	0.477	-20.8	1.519	1.785
29	EECP	no	13	yes, de novo lesion	0.916	0.653	-28.7	0.796	0.301	-62.2	1.839	1.462
30	EECP	no	33	no	n/a	n/a	n/a	n/a	n/a	n/a	n/a	n/a
31	EECP	yes	29	no	1.114	1.404	26	0.813	0.845	4	1.672	1.903
32	EECP	n/d	27	no, non-cardiac exercise limitation	1.727	1.824	5.6	1.459	1.491	2.2	2.687	2.387
33	EECP	yes	26	no	1.322	1.322	0	1.498	1.415	-5.6	1.973	2.037
34	EECP	no	26	no	1.146	1.041	-9.1	1.247	1.512	21.2	1.898	1.924
35	EECP	n/d	22	no	n/a	n/a	-12.8	n/a	n/a	-3.9	n/a	n/a
36	EECP	yes	20	no	1.091	1.230	12.8	1.640	1.708	4.1	1.982	2.179
37	EECP	no	19	no	1.362	1.322	-2.9	1.525	1.550	1.7	1.991	1.964
38	EECP	yes	14	no	0.522	2.183	318	1.041	2.504	93.1	2.173	3.030
39	EECP	no	5	no	1.505	1.051	-30.2	2.033	1.061	-47.8	1.987	2.057
40	None	n/d	36	yes, CABG	n/a	n/a	n/a	n/a	n/a	n/a	n/a	n/a
R1	Rehab	no	106	yes, stent	0.477	0.699	46	0.778	0.653	-16	1.929	1.813
R2	Rehab	yes	68	yes, stent	0.477	0.477	0	1.230	0.845	-31	2.182	2.000
R3	Rehab	no	54	yes, died of heart failure	0.477	0.845	77	1.255	1.267	1	2.041	2.083
R4	Rehab	yes	61	no, non-cardiac death	0.699	0.301	-57	0.477	1.204	152	2.037	2.262
R5	Rehab	yes	112	no	0.301	0.903	200	0.602	0.778	29	1.919	1.940
R6	Rehab	no	1	yes, died from myocardial infarction	0.477	0.778	63	0.477	0.903	89	1.643	2.017
R7	Rehab	no	63	no	0.301	1.415	370	0.845	1.146	36	2.053	2.072
R8	Rehab	no	16	no	1.342	0.929	-31	1.362	0.699	-49	1.978	1.782
R9	Rehab	yes	8	yes, hospitalized for heart failure	0.301	0.954	217	0.602	0.602	0	2.418	2.199
R10	Rehab	yes	13	no	0.301	0.813	170	0.477	0.544	14	2.140	2.249
R11	Rehab	yes	96	no	1.176	1.000	-33	0.477	0.477	0	1.740	1.826

Footnotes:

*excluded from survival analyses due to early withdrawal from study

n/d = not determined due to lack of stress tests

n/a = not available
