# Supplementary material for: Mobilization of Endogenous CD34+/CD133+ Endothelial Progenitor Cells by Enhanced External Counter Pulsation for Treatment of Refractory Angina
Source: Int J Mol Sci. 2024 Sep 18;25(18):10030. doi: 10.3390/ijms251810030 (PMC11432706; doi:10.3390/ijms251810030)
Supplement: Supplementary file 1 [file ijms-25-10030-s001.zip › Table S3 Patient Stress Test Data.pdf]

Patient #	Trtmt	Responded to therapy	pre-DP	post-DP	% Δ DP	pre-TT	post-TT	% Δ TT	pre-CCS	post-CCS	CCS change
1	EECP	no	15246	13650	-10	292	399	37	3	3	0
2	EECP	yes	19360	25200	30	360	550	53	2	1	1
3	EECP	no	13110	16500	26	343	319	-7	3	3	0
4	EECP	no*	15120	No test	n/a	240	No test	n/a	3	3	0
5	EECP	no	23430	22082	-6	360	249	-31	2	3	-1
6	EECP	no	22185	14883	-33	277	236	-15	3	2	1
7	EECP	no	24800	21750	-12	384	386	1	3	1	2
8	EECP	no	26400	18200	-31	586	514	-12	1	1	0
9	EECP	yes	17640	23254	31	330	366	11	3	1	2
10	EECP	yes	15990	16480	3	406	531	31	3	2	1
11	EECP	no	13640	8400	-38	347	205	-41	3	3	0
12	EECP	yes	16800	18837	12	343	377	10	2	2	0
13	EECP	no	30210	28960	-4	345	445	29	3	2	1
14	EECP	yes	8640	12740	47	0	180	100	3	2	1
15	EECP	yes	10780	12600	17	120	229	91	3	2	1
16	EECP	yes	13800	16800	22	180	300	67	3	1	2
17	EECP	no	21250	15540	-27	322	363	13	3	2	1
18	EECP	no	12320	9568	-22	192	324	69	3	2	1
19	EECP	yes	12296	16950	38	466	621	33	3	2	1
20	EECP	no	15600	15400	-1	159	180	13	4	2	2
21	EECP	yes	19320	30400	57	645	661	2	3	1	2
22	EECP	no	20992	18200	-13	370	0	-100	3	2	1
23	EECP	yes	16500	25120	52	484	669	38	3	1	2
24	EECP	yes	20992	18760	-11	540	540	0	2	2	0
25	EECP	no	22540	26350	17	349	425	22	3	2	1
26	EECP	yes	16756	21440	28	330	420	27	3	1	2
27	EECP	no	16660	14690	-12	382	345	-10	2	2	0
28	EECP	no	19376	13965	-28	334	0	-100	4	2	2
29	EECP	no	20091	18271	-9	480	419	-13	4	2	2
30	EECP	no	16912	14980	-11	530	533	0	2	2	0
31	EECP	yes	19360	25840	33	374	447	20	2	1	1
32	EECP	n/d	13090	No Test	n/a	245	No test	n/a	3	1	2
33	EECP	yes	18755	21760	16	366	392	7	2	2	0
34	EECP	no	20720	16770	-19	368	469	27	3	2	1
35	EECP	n/d	No test	No test	n/a	n/a	n/a	n/a	4	3	1
36	EECP	yes	11280	16960	50	167	282	69	3	2	1
37	EECP	no	22400	19740	-12	240	245	0	3	2	1
38	EECP	yes	19040	19360	2	343	421	23	2	2	0
39	EECP	no	16940	19040	12	242	247	2	3	2	1
40	None	n/d	No test	No test	n/a	No test	No test	n/a	n/a	n/a	n/a
R1	Rehab	no	20320	11640	-43	331	251	-24	3	4	-1
R2	Rehab	yes	13920	17250	24	393	398	1	3	3	0
R3	Rehab	no	11830	14250	20	499	251	-50	3	2	1
R4	Rehab	yes	20960	22560	8	459	578	26	1	1	0
R5	Rehab	yes	17640	22560	28	540	719	33	1	1	0
R6	Rehab	no	24640	24000	-3	204	195	-4	1	1	0
R7	Rehab	no	18720	18447	-1	485	556	15	1	1	0
R8	Rehab	no	19584	24800	21	389	360	-7	2	1	1
R9	Rehab	yes	15470	17160	11	408	499	22	3	2	1
R10	Rehab	yes	19680	23920	22	286	332	16	3	1	2
R11	Rehab	yes	19720	22050	12	508	622	22	2	1	1

Footnotes:

*excluded from survival analyses due to early withdrawal from study

n/d = not determined due to lack of stress tests

n/a = not available
