# Supplementary material for: Mobilization of Endogenous CD34+/CD133+ Endothelial Progenitor Cells by Enhanced External Counter Pulsation for Treatment of Refractory Angina
Source: Int J Mol Sci. 2024 Sep 18;25(18):10030. doi: 10.3390/ijms251810030 (PMC11432706; doi:10.3390/ijms251810030)
Supplement: Supplementary file 1 [file ijms-25-10030-s001.zip › Table S4. Patient Clinical Data.pdf]

Patient #	Age	Former smoker	Gender	beta blocker	BMI	Diabetes	Hyper tension	LVEF	PTCA	CABG	clopi-dogrel	Ca blocker	anti-thrombin	pentoxi-phylline	nitrate	diuretic	LDL	HDL
1	64	no	male	yes	19.5	no	yes	0.25	no	yes	no	no	yes	no	yes	yes	75	32
2	n/a	n/a	male	yes	n/a	n/a	n/a	n/a	n/a	n/a	n/a	n/a	n/a	n/a	n/a	n/a	n/a	n/a
3	50	yes	male	yes	30.2	no	yes	0.60	no	no	no	no	no	no	yes	no	n/a	n/a
4	87	yes	male	yes	28.5	no	yes	0.55	yes	yes	yes	no	no	no	yes	yes	84	55
5	73	no	male	yes	28.6	no	yes	0.57	no	no	no	no	no	no	yes	no	100	57
6	80	no	male	yes	29.5	yes	yes	0.55	yes	yes	no	no	yes	no	yes	yes	84	39
7	59	no	male	yes	29.7	no	yes	0.55	yes	no	yes	no	no	yes	no	no	n/a	n/a
8	57	no	male	yes	35.1	no	no	0.60	yes	no	yes	no	no	yes	no	yes	74	40
9	63	no	male	yes	28.2	yes	yes	0.55	yes	yes	yes	yes	no	yes	yes	no	39	31
10	71	no	male	yes	24.7	yes	no	0.55	yes	yes	no	no	no	yes	yes	no	44	53
11	59	yes	male	yes	33.2	no	yes	0.40	yes	no	yes	no	no	no	yes	no	n/a	n/a
12	72	no	male	yes	33.2	no	yes	0.40	yes	no	no	no	no	no	yes	no	68	36
13	72	no	male	yes	33.3	no	yes	0.55	yes	yes	no	yes	no	no	yes	no	89	34
14	65	no	female	yes	40.0	yes	yes	0.35	yes	yes	yes	no	no	no	no	yes	n/a	35
15	80	no	male	no	21.6	no	no	0.25	no	yes	no	no	yes	no	no	yes	84	54
16	62	no	male	yes	32.3	no	no	0.65	no	yes	no	yes	yes	no	no	no	84	50
17	73	yes	male	yes	31.7	no	no	n/a	n/a	no	no	no	no	no	no	yes	90	33
18	71	no	male	yes	23.4	yes	yes	0.20	no	yes	no	no	no	yes	yes	yes	77.2	36
19	64	no	male	yes	33.7	no	no	0.20	yes	yes	no	yes	no	no	no	yes	66	46
20	65	no	male	yes	21.3	no	no	0.19	no	yes	no	no	no	no	no	yes	75	32
21	71	yes	male	yes	30.7	no	no	0.30	yes	no	yes	yes	yes	no	no	yes	63	35
22	61	no	male	yes	33.5	yes	yes	n/a	yes	yes	n/a	yes	n/a	n/a	n/a	n/a	92	39
23	40	yes	male	yes	26.4	yes	no	0.55	yes	yes	yes	yes	no	yes	yes	no	68	42
24	75	yes	male	yes	22.2	no	yes	0.45	yes	yes	yes	no	no	no	yes	no	n/a	n/a
25	60	yes	male	yes	29.2	yes	yes	0.40	yes	yes	no	no	no	no	no	no	n/a	n/a
26	66	no	male	yes	33.5	yes	yes	n/a	n/a	n/a	n/a	yes	n/a	n/a	n/a	n/a	n/a	n/a
27	64	no	male	yes	27.6	no	no	0.55	yes	yes	yes	yes	no	no	yes	no	108	34
28	63	yes	male	yes	41.4	no	yes	0.29	n/a	no	no	yes	no	no	no	no	68	42
29	73	yes	male	yes	22.7	no	no	0.55	yes	yes	yes	yes	no	no	yes	no	39	42
30	53	yes	male	yes	26.3	no	no	0.30	no	yes	no	no	no	no	no	no	n/a	n/a
31	64	yes	male	yes	34.3	no	no	0.55	yes	yes	yes	no	no	no	no	no	83	49
32	83	yes	female	yes	23.2	no	no	0.35	no	yes	no	no	yes	no	no	yes	113	31
33	71	no	male	yes	41.9	no	yes	0.30	yes	no	yes	no	yes	no	yes	no	47	37
34	69	yes	male	yes	32.2	no	yes	0.45	yes	no	yes	yes	no	no	no	yes	68	39
35	87	yes	female	yes	32.1	yes	yes	0.30	no	yes	no	no	no	no	no	no	n/a	n/a
36	n/a	yes	male	yes	25.0	no	yes	0.35	yes	yes	yes	no	n/a	no	no	no	n/a	n/a
37	75	yes	female	yes	28.6	no	yes	0.55	no	no	no	yes	no	no	no	no	n/a	34
38	71	yes	male	yes	33.0	no	yes	0.56	no	yes	no	yes	yes	yes	yes	yes	n/a	n/a
39	n/a	no	male	yes	30.0	yes	no	0.64	no	no	no	no	n/a	no	no	no	n/a	n/a
40	79	yes	male	yes	32.8	no	yes	0.5	yes	yes	no	yes	no	no	yes	no	136	41
R1	72	no	male	yes	25.7	yes	yes	0.7	no	yes	no	yes	no	no	no	yes	59	34
R2	77	yes	male	yes	23.3	no	yes	0.68	yes	no	yes	no	no	no	no	no	61	48
R3	79	yes	female	yes	32.6	yes	yes	0.6	yes	no	no	yes	yes	no	yes	yes	84	57
R4	64	no	male	yes	26.4	no	yes	0.5	yes	no	yes	no	no	no	no	no	121	45
R5	59	no	male	yes	25.8	yes	yes	0.64	no	yes	no	no	no	no	no	no	100	34
R6	68	yes	female	yes	34.4	yes	yes	0.6	no	yes	no	no	no	no	no	yes	114	70
R7	59	no	male	no	27.1	no	no	0.5	no	no	no	yes	no	no	no	no	131	44
R8	65	yes	male	yes	26.5	no	no	0.56	yes	no	yes	no	no	no	no	no	115	49
R9	72	no	female	no	49.7	no	yes	0.56	yes	no	yes	no	no	no	no	yes	110	45
R10	58	yes	male	yes	28.8	no	yes	0.37	yes	yes	no	no	no	no	no	no	79	37
R11	62	yes	male	yes	25.2	no	no	0.6	yes	no	yes	no	no	no	no	no	60	51

Footnotes:

*excluded from survival analyses due to early withdrawal from study

n/d = not determined due to lack of stress tests

n/a = not available
