# Supplementary material for: Mobilization of Endogenous CD34+/CD133+ Endothelial Progenitor Cells by Enhanced External Counter Pulsation for Treatment of Refractory Angina
Source: Int J Mol Sci. 2024 Sep 18;25(18):10030. doi: 10.3390/ijms251810030 (PMC11432706; doi:10.3390/ijms251810030)
Supplement: Supplementary file 1 [file ijms-25-10030-s001.zip › Table S5 Scheme 2007. (v4) Statementí¬checklist of items that should be included in reports of cohort studies..pdf]

Supplemental Table S5

Scheme 2007. (v4) Statement—checklist of items that should be included in reports of cohort studies.

Scheme .	Item #	Recommendation	Reported on page
**Title and Abstract**	1	***(a)*** Indicate the study's design with a commonly used term in the title or abstract	1
		(b) Provide an informative and balanced summary of what was carried out and what was found in the abstract	1
**Introduction**			
Background/rationale	2	Explain the scientific background and rationale for the investigation being reported	2-3
Objectives	3	State specific objectives, including any prespecified hypotheses	2-3
**Methods**			
Study design	4	Present key elements of the study's design early in the paper	2-3
Setting	5	Describe the setting, locations, and relevant dates, including periods of recruitment, exposure, follow-up, and data collection	13
Participants	6	*(a)* Provide the eligibility criteria, as well as the sources and methods of selection of participants; describe follow-up methods	13
Variables	7	Clearly define all outcomes, exposures, predictors, potential confounders, and effect modifiers; provide diagnostic criteria, if applicable	3
Data sources/measurement	8*	For each variable of interest, provide sources of data and details of assessment methods (measurement); describe comparability of assessment methods if there is more than one group	13-14
Bias	9	Describe any efforts to address potential sources of bias	13
Study size	10	Explain how the study size was arrived at	158
Quantitative variables	11	Explain how quantitative variables were handled in the analyses; if applicable, describe which groupings were chosen and why	14-15
Statistical methods	12	(*a*) Describe all statistical methods, including those used to control for confounding	14-15
		(*b*) Describe any methods used to examine subgroups and interactions	14-15
		(*c*) Explain how missing data were addressed	15
		(*d*) If applicable, explain how loss to follow-up was addressed	15
		(*e*) Describe any sensitivity analyses	n/a
**Results**			
Participants	13*	*(a)* Report numbers of individuals at each stage of the study—e.g., number of potentially eligible participants, examined for eligibility, confirmed	3

		eligible, included in the study, completing follow-up, and analyzed	
		(b) Give reasons for non-participation at each stage	3
		© Consider the use of a flow diagram	
Descriptive data	14*	*(a)* Outline characteristics of study participants (e.g., demographic, clinical, social) and provide information on exposures and potential confounders	9, 13
		(b) Indicate number of participants with missing data for each variable of interest	4-5
		© Summarize follow-up time (e.g., average and total amount)	13
Outcome data	15*	* Report numbers of outcome events or summary measures over time	3-9
Main results	16	(*a*) Give unadjusted estimates and, if applicable, confounder-adjusted estimates and their precision (e.g., 95% confidence interval); clearly define which confounders were adjusted for and why they were included in the main results	9
		(*b*) Report category boundaries when continuous variables were categorized	3-9
		(*c*) If relevant, consider translating estimates of relative risk into absolute risk for a meaningful time period	
Other analyses	17	Report other analyses that were carried out—e.g., analyses of subgroups and interactions, as well as sensitivity analyses	n/a
**Discussion**			
Key results	18	Summarize key results with reference to the study's objectives	11-12
Limitations	19	Discuss the limitations of the study, considering sources of potential bias or imprecision; discuss both the direction and magnitude of any potential biases	12
Interpretation	20	Give a cautious overall interpretation of results, considering the objectives, limitations, multiplicity of analyses, results from similar studies, and other relevant evidence	11-13
Generalizability	21	Discuss the generalizability (external validity) of the study's results	12-13
**Other Information**			
Funding	22	Give the source of funding and the role of the funders for the present study and, if applicable, for the original study on which the present article is based	17
